# Resistive Liquid-Vapor Surface Sensors for Liquid Nitrogen and Hydrogen

**DOI:** 10.6028/jres.097.025

**Published:** 1992

**Authors:** J. D. Siegwarth, R. O. Voth, S. M. Snyder

**Affiliations:** National Institute of Standards and Technology, Boulder, CO 80303

**Keywords:** interface, liquid hydrogen, liquid nitrogen, liquid-vapor, resistance thermometer, surface sensor

## Abstract

Ten resistance thermometers were tested as point sensors for detecting the liquid-vapor interface in liquid nitrogen and liquid hydrogen. Test results showed that most could be made to detect the liquid surface and lead orientation can be important. A silicon resistive sensor had the fastest response and produced the greatest signal change.

## 1. Introduction

In the early 1960s a number of liquid-vapor (L-V) interface sensing devices were tested in liquid hydrogen (LH_2_) [1–3]. Part of this work was done at the National Institute of Standards and Technology (then the National Bureau of Standards) for NASA Lewis Research Center. The sensors tested were then commercially available and had various principles of operation. Differences in resistance, capacitance, light reflection, acoustic impedance and viscous damping indicated whether the sensors were in liquid or gas. Since many of the sensors in these early tests were liquid level measuring devices, resolving the liquid surface well in the vertical direction was all they were required to do. Many new level gaging devices are commercially available today but like the sensors examined in the earlier tests, they are still mostly uniaxial devices. Often, the sensing element itself is large in the plane perpendicular to the vertical axis.

The objective of this study was to identify devices to serve as L-V detectors in zero gravity. In zero gravity, the liquid-vapor interface can be moving in any direction. This means the L-V sensor must approximate a point sensor to give the same resolution of the passing of an interface from any direction. Resistance thermometer and optical type sensors approximating point sensors were the only sensors tested in this work. The results of the tests of resistive sensors are reported here. The optical sensor tests results have been reported elsewhere [4].

A resistive L-V sensor has a temperature dependent resistance and when operated at low power dissipation, can serve as a thermometer. The heat transfer from the sensor to the surroundings must be sufficiently large that the temperature difference between the thermometer and the surroundings is below the tolerable measurement error. The difference in the heat transfer from the thermometer to a liquid relative to the heat transfer to equilibrium vapor can be large. A thermometer thus becomes an L-V sensor when the power is raised until the sensor temperature difference between liquid and vapor, hence the resistance difference, becomes large enough to detect easily.

A small sensor size is advantageous not only for spatial resolution but also for sensor response time. The smaller the mass and surface area, the faster the response upon leaving the liquid. The larger d*R*/d*T*, the greater the signal change. Some overlap of these two effects occurs because they are coupled through the magnitude of the temperature change between entry and exit of the liquid. A small mass allows the sensor to respond more rapidly when the sensor enters the liquid. A small sensor surface area however, which is an advantage when the sensor leaves the liquid, slows the response when the sensor enters the liquid.

Since the work of the 1960s, a number of new, miniature resistance thermometers have become available. Many of the resistive L-V sensors tested in this work are commercially available thermometers operated at much higher power than specified by the manufacturer. The time constants and magnitude of the output signal change have been examined over a range of power levels for both liquid hydrogen and liquid nitrogen (LN_2_).

## 2. Apparatus

**Fig. 1:**
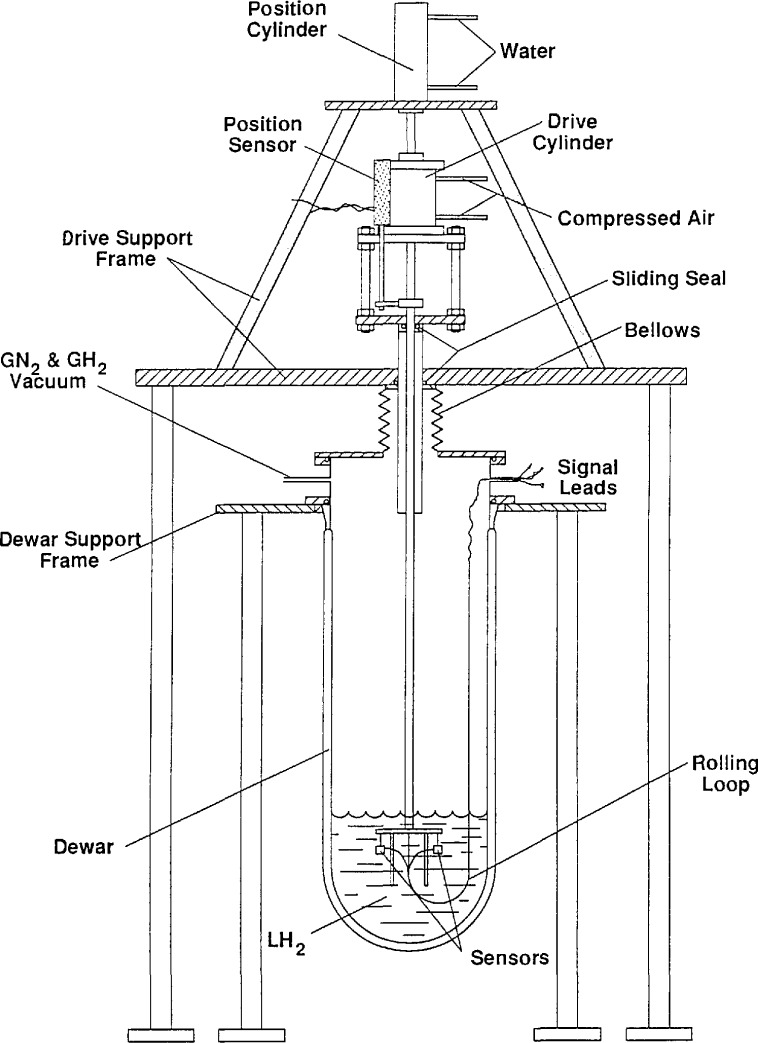
Test apparatus for liquid-vapor surface sensors.

The apparatus used to test the sensors is shown in Fig. 1. The testing in LH_2_ was done in a sealed glass Dewar system to prevent air from contacting the hydrogen. Up to six sensors could be mounted on a holder and tested simultaneously. The holder was cycled rapidly up and down by the manually controlled drive cylinder, which was a double acting air cylinder. The total travel was about 10 cm. Air cushions in each end of the cylinder brought the piston to a stop at the end of its travel. The water driven cylinder served to adjust the vertical position of the drive cylinder and sensor holder so that the sensors passed through the liquid interface somewhere between the 2.5 and 7.5 cm positions of the drive cylinder travel. A linear potentiometer connected to the drive cylinder shaft gave a position signal for which 1 V was equivalent to 2.5 cm of travel. The position at which the sensors crossed the interface could be determined either by placing a sensor at the liquid level and reading the position voltage or by noting the voltage at which a faster L-V sensor started the transition between the gas signal, *S*_g_, and the liquid signal, *S*_l_.

The velocity of the sensor holder at mid stroke was about 3 m/s at the drive air pressures used. The transit time of a sensor through the liquid interface was less than 2 ms for most of the sensors tested. The shortest cycle times of the sensor holder were about 1.5 s.

Individual sensors were mounted on stainless steel blades, 51 × 17 × 0.5 mm in dimension. The sensors were centered in 7 mm diameter holes punched near the end of these blades. With a few exceptions, the sensors were supported by their leads. The sensor leads were either clamped mechanically or cemented to the blades. The blades were bolted to the sensor holder at the ends opposite the sensors. Most of the tests were done with the blades mounted so the leads to the sensor were horizontal. One test each in LN_2_ and LH_2_ was done with the blades mounted so the leads ran vertically down to the sensors.

The electrical leads to the holder were fastened to and guided by a thin steel strip which was attached on one end to a fixed point at the dewar wall and to the moving sensor holder on the other. The rolling loop in this strip allowed the leads to follow the sensor holder.

The sensors were powered either by a constant current or a constant voltage source. The sensor output was the voltage across the sensor for constant current, or the voltage across a fixed resistor in series with the sensor for constant voltage. The constant voltage mode was chosen when the voltage needed to supply the desired power exceeded 10 V or because of the temperature dependence of the sensor resistance was positive.

The output signals from the L-V sensors and the position sensor were sequentially read by a 13 bit high speed 8 channel multiplexer and analog to digital converter card in a laboratory computer. The card was capable of reading at a rate near 10^5^ readings/s. Since the reading rate never exceeded 2000 readings/s for one channel, no correction for channel delay was needed. The elapsed time for each test run was limited by the amount of data that could be stored. When only two data channels were recorded, 13,000 readings for each channel was the maximum. The measurement time was added to the data files after the data were taken by multiplying the reading number by the reading period.

The response time of the sensor was defined in these tests as the time elapsed between the time the sensor crossed the liquid surface according to the position sensor voltage and the time at which the L-V sensor signal, *S*, reached

$$S=\left(S_{l}-S_{g}\right)/2.$$ (1)

Semiconductor sensors have resistances that increase with decreasing *T* so *S*_l_*>S*_g_ for constant current measurements. Shorter response times than those determined from the *S* of Eq. (1) could be obtained if the signal processor used a higher sensor signal level to indicate entering the liquid surface and a lower one to indicate leaving the surface. The data were analyzed by the computer to determine the response times.

The sensors were cycled in and out several times in these tests. Because the test duration was limited by the amount of data that could be stored, the reading frequency was adjusted to the minimum frequency that could resolve the most important of the signal details. The slowest response time was generally the detail of interest and that was the response time in switching from liquid to gas. The response time from gas to liquid of most sensors was much shorter and was not resolved more accurately, since the response time was less than the measuring period.

## 3. Resistive Sensors Tested

A brief description of the resistive sensors tested is given in below and in Table 1.

LVD Goddard (LVDG): These sensors were built at Goddard Space Flight Center. The sensor consisted of a small cube of doped silicon with the two leads attached to opposite faces. The design was that used for L-V interface sensors in the SHOOT experiment [5]. The doped silicon chips used in these tests were selected by the Goddard staff for high sensitivity at 20 K (LH_2_ temperature at 101 kPa).

Silicon-on-sapphire (SOS): This sensor was built at NIST for use as a fast response thermometer.

K: This sensor is a commercially available thermometer. It was designed to operate over the range where the resistance (*R*) versus temperature (*T*) has a positive slope. A minimum in the *R* versus *T* curve occurred at about 100 K. A little below LN_2_ temperature (77 K at 101 kPa) *R*, measured by a digital multimeter, exceeded 25 MΩ. This sensor proved to be too fragile to be supported only by its leads. After two sensors so supported broke, a sensor was cemented to some stainless steel shim stock about 0.1 mm thick.

PT1 and PT2: These sensors are commercially available platinum thermometers consisting of platinum films on ceramic substrates. They differ mainly in physical size and resistance. The resistance at 0° C of the smaller is 100 Ω and the larger is 1 k Ω.

DT0, DT1, and S: These three diode thermometers came from a commercial source. DT0, essentially the same thermometer as DT1 but in a different envelope, is no longer made. DT1 and S were built by two different manufacturers but have very similar voltage versus temperature responses. They differed mainly in that DT1 was encapsulated, while *S* was not.

Mdac: This sensor was a 12 kΩ, 1/4 W resistor in parallel with a 1 kΩ nominal thermistor of unknown manufacture. This sensor is used by an aerospace company both as a L-V sensor and as a thermometer. Two units were tested in this work. One consisted of the bare thermistor and resistor on the end of a probe and connected to small gage copper leads. The other unit consisted of the two elements epoxied together with their leads soldered to two heat sink plates about 7 × 33 ×1/2 mm in dimensions.

CARBONl: This carbon resistor was of unknown manufacture but had sufficient temperature dependence to be a usable carbon thermometer.

## 4. Test Results

### 4.1 LVD Goddard, LH_2_ Tests

**Fig. 2:**
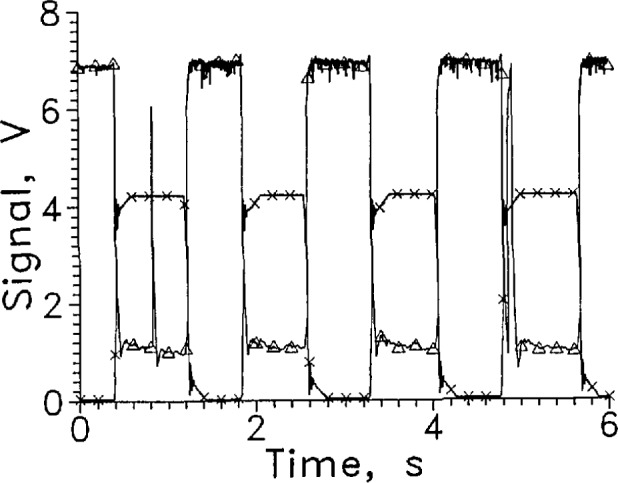
Output signal from LVDG cycled between LH_2_ and GH_2_, A. Position sensor voltage, ×. Leads horizontal, *I* = 9 mA, every 100th data point shown. For this and all subsequent graphs of signal versus time, the sensor is in the liquid when the position sensor voltage reading is less than 1 V and out of liquid when the reading is above 3 V.

Figure 2 shows several cycles of a rapid cycle test of LVDG in LH_2_ at 9 mA constant current. A large signal level change was obtained. The response time for entering and leaving cannot be distinguished from zero on this time scale.

Figures 3a and 3b show the first withdrawal from the liquid and return to the liquid from the same data as Fig. 2 on an expanded time scale. The L-V sensor level defined by Eq. (1) was 3.95 V, and occurred at 0.4090 s. The L-V sensor actually crossed the liquid surface at 0.4075 s, when the position sensor output was 1.9 V. The difference of 1.5 ms was the response time. The slope of the position sensor curve at 1.9 V gives a velocity of the sensor through the liquid surface of over 3 m/s which means the LVDG sensor passed through the liquid surface in less than 0.1 ms. The oscillation in the signal from the position indicator at the end of the holder travel was caused by the air cushion at the end of the drive cylinder stroke.

When the sensor moved from the gas to liquid, Fig. 3b, the sensor voltage at constant current started increasing as soon as the sensor started moving in the gas. The sensor arrived at the liquid surface (1.9 V on the position sensor) at 1.2265 s. The sensor signal (3.95 V) indicating passage through the liquid surface, occurred about 2 ms later.

For the dozen cycles of the test for which four cycles are shown in Fig. 2, the average response times entering and leaving the liquid were 1.5 and 3.5 ms, respectively. The signal level change for this sensor was large at a constant current of 9 mA, changing from about 1 V in the gas to 7 V in the liquid.

The sensor signal should remain stable at the liquid value in liquid or the gas value in gas. LVDG occasionally made transitions to the opposite state without sensor movement. Occasional spikes appear in the sensor signal shown in Fig. 2 that are not associated with transitions through the liquid surface. The upward spikes when the sensor was in the gas may result from splashing from the sensor holder passing through the liquid surface. The downward spikes when the sensor was in the liquid may be caused by splashing, but they may also have resulted from momentary transitions to film boiling on the sensor surface. Considerable splashing occurred both when the sensor holder entered and left the liquid as could be seen through the slits in the silvered Dewar walls.

**Fig. 4:**
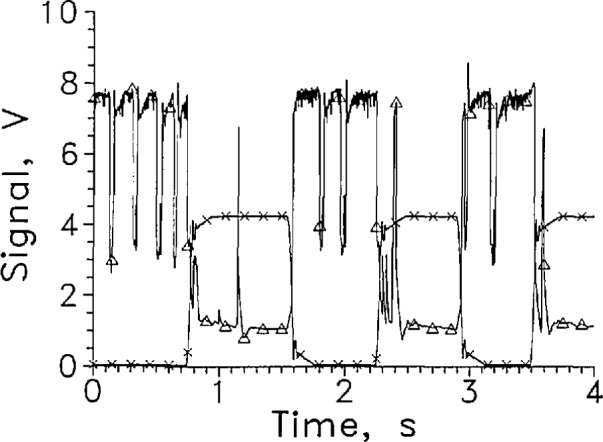
Output signal from LVDG cycled between LH_2_ and GH_2_, Δ. Position sensor voltage, ×. Leads horizontal, *I* = 11 mA, every 75th data point shown.

**Fig. 5:**
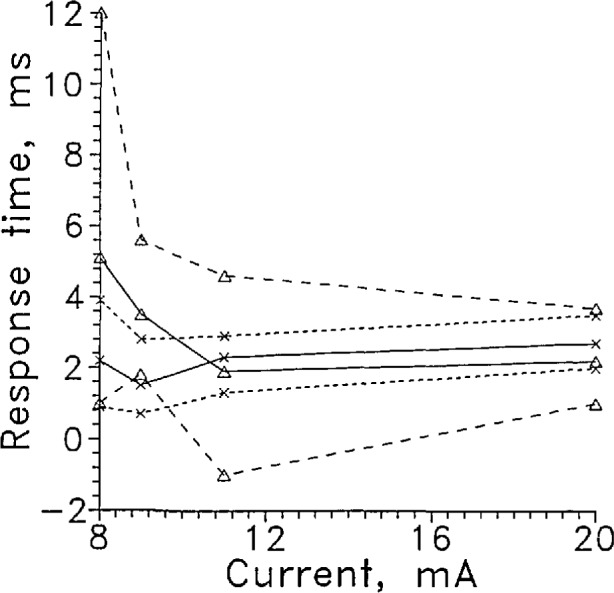
Averaged response time of LVDG as a function of current for the sensor entering the liquid, ×; leaving the liquid, Δ. Dotted lines show total data scatter.

When the sensor current was raised to 11 mA, the sensor voltage in the liquid becomes quite unstable, as shown in Fig. 4. At 20 mA, the in-liquid signal level decreased to less than 4 V (see Fig. 13) and was noisy in the liquid. The voltage in the gas increased to about 1.5 V. The stability of the sensor signal and the amount of signal change decreased while the response time returning to the liquid increased slightly, Fig. 5. The solid lines in Fig. 5 show the response times averaged over about 10 transitions as a function of sensor current. The dotted lines show the envelope of the fastest and slowest responses at each current. The sensor at high currents apparently heated so much above liquid temperature that a longer time was required to cool it back to liquid temperature. When the current was lowered below 9 mA, the response time leaving the liquid increased rapidly as Fig. 5 shows.

**Fig. 6:**
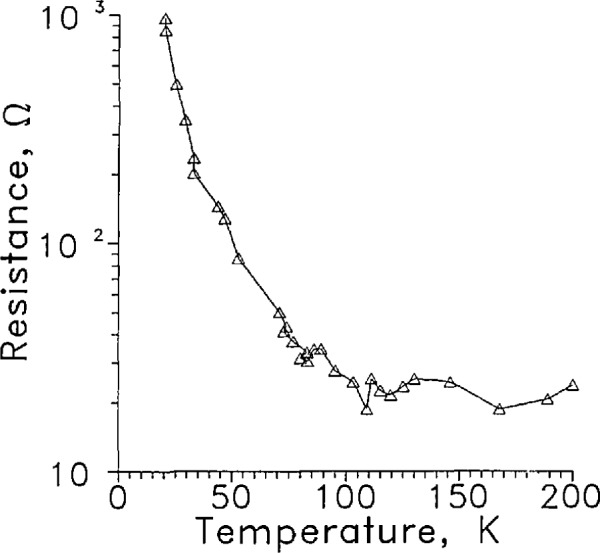
Resistance of LVDG as a function of temperature, Δ. Leads horizontal. *I* = 10 μA.

The large change of the sensor signal between liquid and gas and the rapid response to the surface crossing suggests that the sensor resistance, *R*, is a strong function of temperature, *T*, around 20 K. A measurement of *R*(*T*) between 200 and 20 K is shown in Fig. 6. The sensor current was 10 μA, well below the level causing self heating. The scatter in the data between 80 and 120 K probably was caused by low resolution of the signal processing electronics at the low signal level. The slope, d*R*/d*T* was large at 20 K as anticipated. The LVDG sensor heated less than 50 K in gas to achieve the 7 to 1 signal level change.

**Fig. 7:**
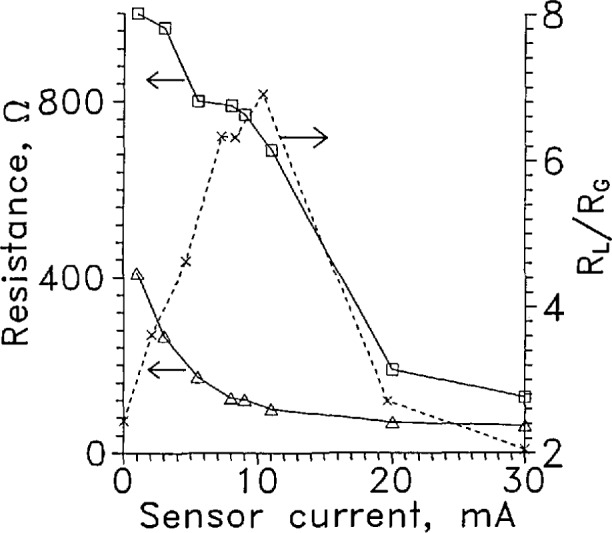
Resistance of LVDG as a function of current, in GH_2_, Δ; and in LH_2_; □. *R*_l_/*R*_g_ ×. Leads horizontal.

Figure 7 shows the resistance of the sensor in gas and in liquid as a function of the sensor current at currents in the L-V sensor operating range. The resistance in the gas decreases rapidly with increasing current at lower currents but levels out above about 10 mA as would be expected for an *R*(*T*) dependence like that shown in Fig. 6. The resistance in the liquid decreases rapidly above about 10 mA current. The decrease of *R* with increasing sensor current for the sensor in LH_2_ results from the increase of temperature needed to transfer the sensor power to the liquid. Above about 10 mA, the heat flux to the LH_2_ was sufficient to cause film boiling on the sensor surface which reduced the heat transfer coefficient. This caused the sensor temperature to increase and *R* to decrease. From Fig. 4, the sensor became a thermoelectric oscillator at 11 mA constant current in liquid. The instability follows from the strong inverse relationship between power level and temperature at constant current.

The ratio of the resistance in liquid to the resistance in gas is also shown in Fig. 7. This ratio was highest at 11 mA, where the sensor oscillation was first seen. The sensor resistance in liquid was only about twice that of gas at 30 mA constant current. The speed and stability was best in these tests for a current around 9 mA for an LVDG sensor of this size, sensitivity and mounting. Any change from this configuration would require a redetermination of the best operating current.

In the first tests in this series, the cycle rate was about one every 4 s and the measurement resolution was 0.1 s. At that rate the response of the LVDG sensor showed only infrequent signal spikes not associated with an actual transition. This suggests that the number of extraneous spikes observed increases at faster cycle times.

To reduce the splashing caused by the sensor holder, the sensor holder was redesigned. The blades to which the sensors were attached were mounted vertically. As a result the sensor leads now ran vertically down to the sensor. The average response time for LVDG in this configuration for entering the liquid was unchanged from that with the leads horizontal. However, in five of the ten cycles recorded at 1 kHz reading rate, an upward spike in the LVDG signal to the liquid reading occurred (Fig. 8a at 9.26 s), between the start of the sensor motion and the entrance to the liquid. In all but one of the remaining five tests, a precursor to the main transition of LVDG was seen. A small precursor occurred at 5.66 s (Fig. 8b).

The average response time of LVDG leaving the liquid increased to almost 21 ms. Figs. 8a and b. The 21 ms response time was measured to the first response of the sensor to vapor. On about half the liquid-to-gas transitions, the LVDG signal cycled between liquid and gas for about 0.1 s after LVDG left the liquid. Fig. 8a. On the remaining half of the test cycles, the LVDG signal cycled back to the liquid reading as much as 0.3 s after the sensor departed the liquid. Fig. 8b.

We believe the increase of the liquid-to-gas response time and the cycling after the initial response was caused by liquid draining down the leads to the sensor from the holder. This source was probably enhanced because the insulation over the leads, which consisted of varnished paper, had partially detached allowing liquid to enter the crack between it and the stainless steel blade holding the sensor.

The starting position of the sensor relative to the liquid surface appeared to cause differences in the response time when the leads were vertical. The measuring rate of 50/s was too low to measure the gas-to-liquid response accurately but the liquid-togas response time appeared to be 5 to 6 ms longer when the sensor holder had completed about 80% of its travel before the sensor left the liquid. The difference in the amount of splashing is the presumed cause. The in-liquid voltage was somewhat larger at a given sensor current than the voltage obtained with horizontal sensor leads.

The sensor holder with the leads vertical was built to reduce splashing in the hope that it would help to eliminate signal transitions that occurred without the sensor crossing the liquid boundary. The performance was definitely poorer than achieved with the original holder. Based on the difference of the test results from the two configurations tried so far, further study of mounting effects would be useful.

### 4.2 LVD Goddard LN_2_ Tests

From Fig. 6, it is clear that d*R*/d*T* for LVDG at LN_2_ temperature (77 K) was small compared to the slope at 20 K. The resistance only changes from about 40 Ω at 77 K to 20 Ω at ambient. For a four lead measurement, *R*_l_/*R*_g_, which is *S*_l_/*S_g_* at constant current, could be no more than 2. Since we made the measurements with two leads, the 35 Ω leads reduced *S*_l_/*S_g_* to no more than 4/3.

**Fig. 9:**
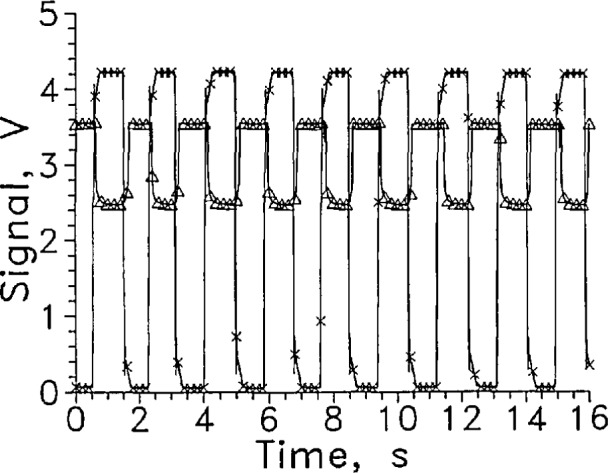
Output signal from LVDG cycled between LN_2_ and GN_2_, Δ. Position sensor voltage, ×. Leads horizontal, *I* = 50 mA, every 100th data point shown.

A rapid cycle test of LVDG is shown in Fig. 9 for a 50 mA sensor current. The output signal at this time resolution still approximates a square wave. Unlike the LH_2_ tests, no spikes occurred in the LN_2_ tests signifying no temporary return to the previous state.

The response times were much longer for LN_2_, Table 2, than for LH_2_ probably because of the higher sensor heat capacity. The preferred operating current was 50 mA since the liquid-to-gas response was equal to the gas-to-liquid response. Also, the sum of the response times for one cycle was less for 50 mA than for 40 mA.

The liquid-to-gas response time for the LVDG at 40 mA varied more than at 50 mA, as shown in Table 2. The gas-to-liquid response time scatter was much less at both currents than the liquid-to-gas response time scatter. The greater variation in the liquid-to-gas response times probably was caused by varying amounts of liquid retained on the sensor though splashing of the liquid when the holder departs the surface may also contribute.

A difference in the responses of the sensor with the different positions of the sensor holder relative to the liquid surface was observed for the 40 mA results as Table 2 shows. The splashing was less when the liquid level was high, but the liquid-to-gas response time was still slower. In one test the sensor failed on some cycles to respond before the sensor returned to the liquid, indicating a response time as long as 1.5 s. At 50 mA, no difference in response time with liquid height was evident.

### 4.3 K and SOS Sensors

The K and SOS sensors are doped silicon films rather than bulk doped silicon like LVDG. The films were supported on sapphire substrates. The K sensor substrate was so thin that it required support. The doping level was high to give good sensitivity around ambient temperatures. The resistance went through a minimum around 130 K and then rose rapidly below 77 K and became essentially infinite well above 20 K, which rendered the sensor unusable as an L-V sensor for LH_2_.

**Fig. 10:**
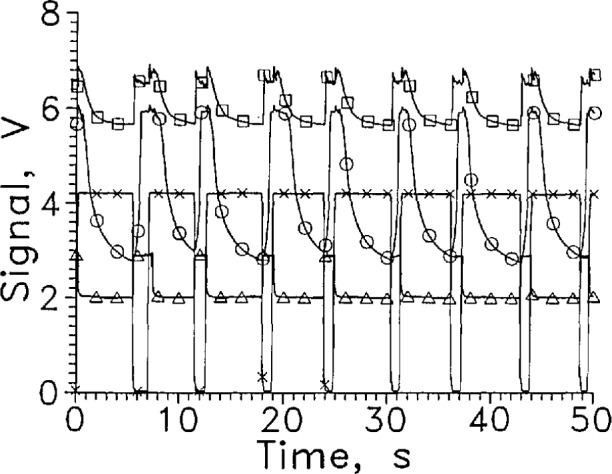
Output signal from LVDG at 40 mA, Δ; K at 5 mA, □; and PT1 at 20 V, O; cycled between LN_2_ and GN_2_. Position sensor voltage, ×. Leads horizontal, and every 100th data point shown.

For LN_2_, the K sensor gave a 5% change in signal level at 5 mA (10 mW) and had a response time of about 1 s when the sensor left the liquid. The output signal obtained in one test is shown in Fig. 10. When the sensor entered the liquid, the response time was 50 ms or less. The 20 Hz signal reading frequency of the fastest test did not resolve the response time to better than 50 ms. At currents of 6 mA and above, the sensor temperature increased sufficiently to reach the high temperature side of the resistance minimum when the sensor remained out of the liquid for a few minutes. This caused the sensor to respond upon reentering the liquid by a voltage decrease instead of an increase. For short periods out of the liquid, the sensor did not heat above its resistance minimum and responded with a voltage increase as at 5 mA current.

The K sensor was tested at 77 K and below at least three times with many repeated tests each time. The sensor was cemented to 0.1 mm shim stock to prevent breakage.

The SOS sensor in LH_2_ at 60 mA constant current (0.4 W) showed a signal level change of more than 30% between liquid and gas. The response time of the sensor leaving the liquid was about 1.5 s for SOS I which had an approximately 1 cm^2^ substrate. SOS II had the excess substrate trimmed off and smaller leads attached. The liquid-to-gas response time for SOS II was just under 1 s. The SOS II response time upon re-entering the liquid was no more than 0.1 s.

In LN_2_, the change of the resistance of SOS II was only about 5% from gas to liquid. The response time of SOS II was rapid upon entering the LN_2_ but the response time to removal from the liquid was as long as 9 s. A lead failed when the current was increased above 60 mA, and no further tests were carried out. SOS II had the disadvantages that the sensor is not commercially available, the response time was slow, and the signal change was small.

Neither the SOS nor the K sensor were tested for effects of lead orientation.

### 4.4 Thin Film Pt Thermometers

The Pt film thermometers tested have the advantages of being commercially available and inexpensive. PT1 and PT2 had ambient resistances of 1 Ω and 100 Ω, respectively.

**Fig. 11:**
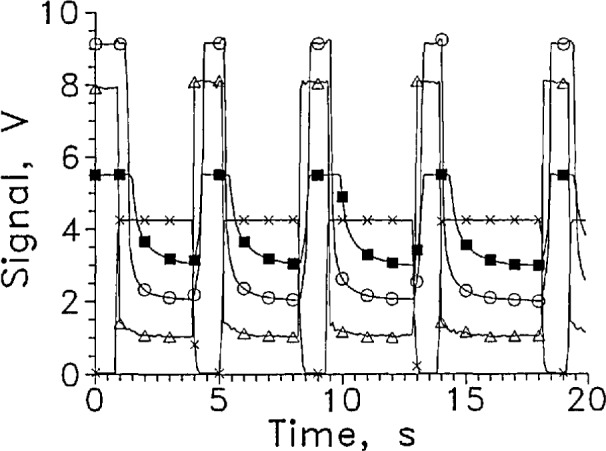
Output signal from LVDG at 9 mA, Δ; PT1 at 20 V, O; and PT2 at 12 V, ■; cycled between LH_2_ and GH_2_. Position sensor voltage, ×. Leads vertical, and every 100th data point shown.

For LH_2_ tests, these two sensors were mounted only with the leads vertical. Figure 11 shows an example of one test of PT1 and PT2 in LH_2_. The sensors were powered by constant voltage sources, and the series resistances used are given in Table 3. The best performances obtained are given in the table. The size of the signal change between entering and leaving liquid was unexpected since Pt thermometers lose sensitivity below 20 K.

The liquid-to-gas response of PT2 was slower than that of PT1. In a later test, PT2 failed to respond for more than 3 s when withdrawn from the liquid. The sensor was returned to the liquid before the gas response occurred. The reason for the slower response for a smaller sensor is not known.

**Fig. 12:**
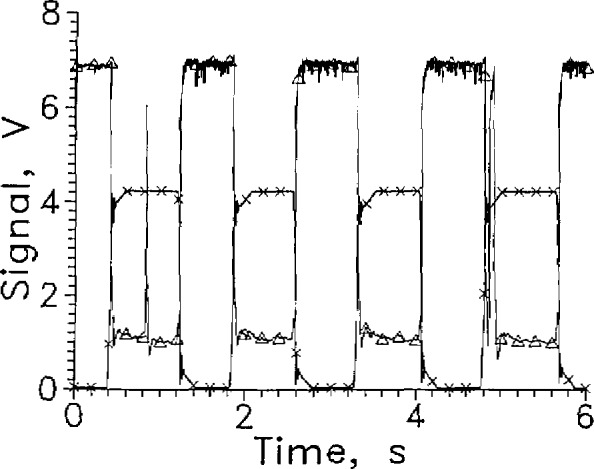
Output signal from LVDG at 40 niA, Δ; PT1 at 20 V, O; and PT2 at 10 V, ■; cycled between LN_1_ and GN_2_. Position sensor voltage, ×. Leads horizontal, and every 10th data point shown.

Results from a test of PT1 and PT2 in LN_2_ are shown in Fig. 12. The signal voltage ratio, *S*_l_/*S*_g_ is at least 2 at the cycle rate shown. The ratio is about 2.5 for a slower cycle time. Table 4 gives some characteristics of PT1 in LN_2_ for three different voltages. The liquid entry and liquid exit response times of PT1 were closest to equal at 20 V. The percentage scatter of the response times was least for PT1 at 20 V. The signal level change was greater for PT1 in LN_2_ than that obtained for LVDG, Fig. 10. The response time was slower than that of LVDG and the power is higher, both of which would be expected because PT1 is larger. The PT1 sensor can be used for LN_2_ surface sensing when fast response is not needed.

When the LN_2_ tests were carried out with the leads extending vertically above the sensor, the liquid exit average response time at 20 V increased to about 2 s while the liquid entry time remained unchanged. This lengthening of the response time is again attributed to liquid draining off the sensor holder down the leads to the sensor.

The performance of PT1 in LN_2_ was better in terms of signal magnitude than for the smaller sensor PT2, Fig. 12. The response times of PT2 were similar to those of PT1 even though we expected they would respond more quickly because of its smaller surface area and size. The response time of PT1 was often faster than PT2 when entering the liquid which was not expected, but was usually slower upon leaving it. PT2 was only tested in the vertical lead configuration. The long decay of the signal level when the sensor leaves the liquid makes the sensor response time highly sensitive to the voltage level chosen to signify the completion of the transition.

The large signal change and the commercial availability of these sensors make them attractive if the high power dissipation and the slow response are acceptable to the application.

### 4.5 Diode Thermometers

**Fig. 13:**
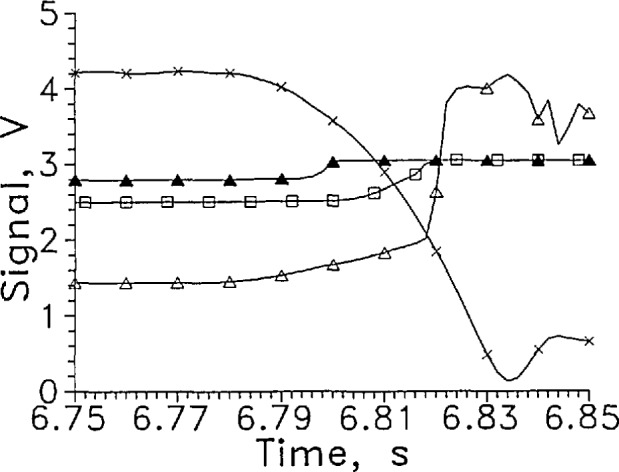
Output signal from LVDG at 20 mA, Δ; DT1 at 40 mA, ▲; and *S* at 40 mA, □; for one transition from GH_2_ to LH_2_. Position sensor voltage, ×. Leads horizontal, and eveiy 5th data point shown.

Some test results for the DT1 and S diode thermometers in hydrogen are shown in Fig. 13. The output signal was the voltage required to maintain a constant current of 40 mA through the sensors. The response time was short for these sensors entering the liquid. In some cases the signal response preceded the time when the sensors entered the liquid. The three sensors of Fig. 13 were all in the same horizontal plane and entered the liquid within about 3 ms of 6.817 s. The early response of the diodes may have been caused by splashing when the holder enters the liquid. Increased cooling due to the motion of the sensor while still in the gas may have caused the early transition by the sensor.

**Fig. 14:**
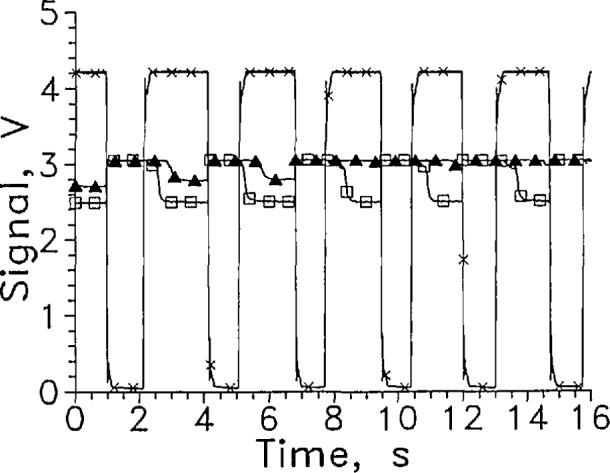
Output signal from DT1 at 40 mA, ▲; and *S* at 40 mA, □; cycled between LH_2_ and GH_2_. Position sensor voltage, ×. Leads vertical, and every 300th data point shown except for DT1 which is 305.

**Fig. 15:**
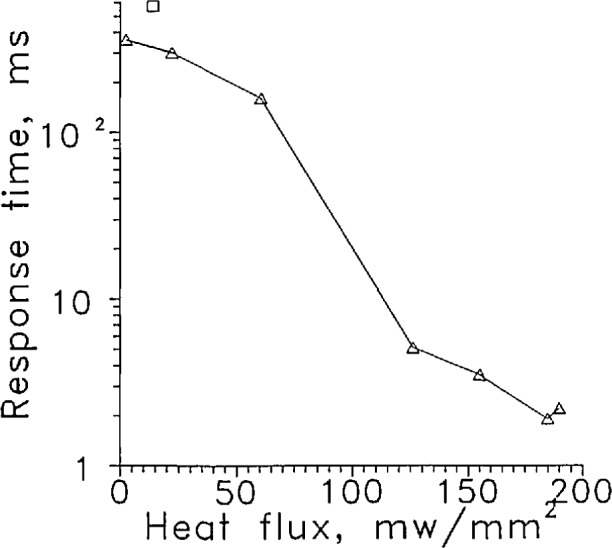
Response time as a function of heat flux for LVDG; Δ; for *S* diode, □.

Since the response time of the diodes was so much more rapid entering the liquid than leaving, Fig. 14, a higher power should be used. Figure 15 shows a plot of the response time leaving the liquid as a function of the estimated heat flux for LVDG and a data point for the *S* diode. At heat fluxes below 150 mW/mm^2^ the response time is strongly dependent on flux. This curve can be used to estimate the power needed by a sensor with a different surface area if the liquid retained on the surface is the dominant factor in the response time. A data point for the highest power to the *S* diode is shown. The *S* sensor had a response time twice as long as LVDG at the same flux. The surface area per unit volume of the *S* diode is about half that of LVDG. The longer response time suggests a contribution from the sensor heat capacity. About 10 times more power would be needed to raise the *S* sensor heat flux up to the 125 mW/mm^2^, which should reduce its response time to less than 10 ms. Alternatively, the surface area of the diode could be decreased to raise the heat flux. The manufacturer of one diode sensor suggested that the current not exceed 10 mA. Current above 40 mA was not used for fear of destroying it.

Since the unencapsulated *S* thermometer had an area about 2.5 times smaller than the encapsulated DT1, the *S* sensor should heat to a higher temperature in the gas for the same current. The signal voltage should then be lower for *S* in the gas and it was. When the *S* diode was in liquid, however, no heating of the sensor was observed.

The smaller surface area and size of the *S* diode allowed it to respond more quickly than DT1 upon withdrawal from LH_2_. The response time for DT1 to withdrawal from LH_2_ was long enough that it did not always have time to respond on every cycle before the sensor re-entered the liquid, as shown in Fig. 14. Again, higher currents should speed the out-of-liquid response of these sensors if they are not damaged by higher power dissipation. Higher current should also increase the *S*_l_/*S*_g_ ratio which was about 1.2 at 40 mA.

The change of the signal level between LN_2_ and GN_2_ was so small for the *S* sensor that it was comparable to the noise in the signal channel. The in-to-out response was several seconds. The current through DT1 and *S* must be much greater than the 40 mA maximum current used in these tests if a satisfactory performance as a surface sensor is to be achieved in LN_2_.

The DT0 sensor response upon withdrawal from the LH_2_ was somewhat faster than DT1. The diodes were not tested with the leads in the vertical orientation.

### 4.6. Mdac Sensor

**Fig. 16:**
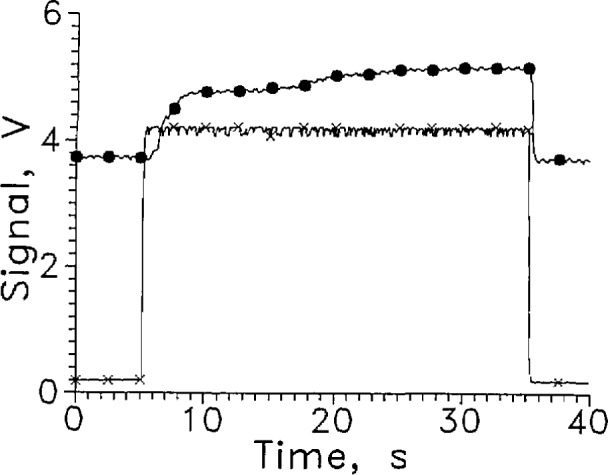
Output signal from Mdac for one cycle between LH_2_ and GH_2_, ●. Position sensor voltage, ×. Leads horizontal, *V*= 100 V, every 25th data point shown.

One test of the Mdac unit attached to heat sinks is shown in Fig. 16 for LH_2_. The sensor responded in a few tenths of a second upon entering the liquid. The response was slow upon leaving the liquid and the noise level was high relative to the signal change until the voltage on the sensor was well in excess of 50 V. The response time leaving the liquid was about 2 s at 100 V.

**Fig. 17:**
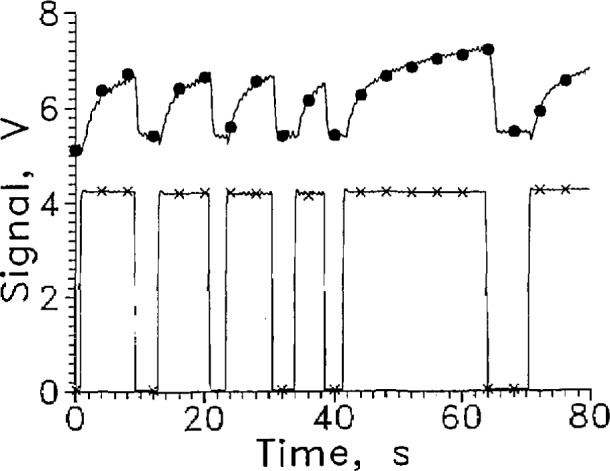
Output signal from Mdac cycled between LN_2_ and GN_2_, ●. Position sensor voltage, ×. Leads horizontal, *V*=100 V, every 20th data point shown.

For liquid vapor surface sensing at 77 K and below, the thermistor is superfluous because its resistance is so high that the carbon resistor carries all the current. Voltages above 100 V were not tried to avoid breakdown of the insulating varnish on the leads. The resistor should be reduced from 12 kΩ to perhaps 1 kΩ to reduce the required voltage. The dependence of *R* on *T* for a carbon resistor varies with manufacturer. A brand whose *R* has a strong dependence on *T* should be selected. When the Mdac with heat sinks was tested in LN_2_, either capacitance effects or momentary shorts in the leads caused signal spikes when the sensor holder moved. A Mdac sensor without heat sinks but with heavier insulation on the leads was tested in LN_2_ in a similar test system external to the gas tight dewar assembly of Fig. 1. Test results with 100 V applied are shown in Fig. 17. The response time leaving the liquid was about 2 s. The response time entering the liquid varies from about 0.2 to 1.2 s depending on how long the sensor was out of the liquid. The signal amplitude changed from about 5.2 to 6.7 V for a short cycle time. As Fig. 17 shows, the sensor does not reach equilibrium after withdrawal from the liquid even after 20 s. A lower nominal resistor operating at constant current should reach equilibrium more rapidly. Grinding the shell off the resistor should also speed the response.

### 4.7 Carbonl

For the last LN_2_ and LH_2_ tests a 925 Ω, 1/8 W carbon resistor (Carbonl) was tested. The signal level change of this resistor was less than for Mdac and the response time similar.

The output voltage change for Carbonl for hydrogen was from 2.8 to 3.6 V. The signal ratio was about 1.2 for 25 V across the sensor and a 200 fi resistor in series. The out-to-in response time was about 60 ms. The in-to-out response was about 0.5 s. Both response times increased with increasing current. It is not understood why this should happen on the out transition. It is clear that a carbon resistor with a larger dependence of resistance on temperature is required for the performance of carbon resistors to be satisfactory, Carbonl did respond faster than Mdac as would be expected because it is smaller,

## 5. Discussion

An ideal L-V sensor could be described as follows: the signal change should be at least a factor of 10 larger than the combined noise level, drifts of the power supply and drifts of the signal processing electronics; the response time should be less than 10% of the fastest response time required for the measurement and the sensor should not perturb the liquid surface as it passes through.

The response time of a resistive sensor leaving the liquid depends on the integral of the sensor heat capacity between the sensor temperature in the liquid, *T*_l_, and the sensor temperature in the gas, *T*_g_, the amount of liquid in the residual film carried out on the sensor surface, and the power dissipated in the sensor. The amount of liquid in the residual film should be primarily a function of the surface area of the sensor.

The response time of the sensor entering the liquid, however, depends only on the integrated heat capacity of the sensor between *T*_g_ and *T*_l_ and the surface area through which heat transfers to the liquid.

An ideal resistive sensor should thus consist of the smallest practical resistive element. Any inactive material in the sensor increases the power density in the active part needed to cause a given temperature change. Making the sensor smaller reduces its heat capacity which decreases the response times entering and leaving the liquid. Making it more compact in terms of area decreases the leaving response time. A smaller surface area for the same volume increases the entering response time because the smaller surface reduces the heat transfer rate to the liquid.

Increasing d*R*/d*T* of a sensor at a fixed *T*_g_ − *T*_l_ increases the signal level change, *S*_g_ − *S*_l_. Conversely, at constant *S*_g_ − *S*_l_, *T*_g_ − *T*_l_ is decreased as d*R*/d*T* increases thus the amount of heat transferred to or from the sensor in moving between the two states is reduced. In the latter case, the response times both entering and leaving the liquid decrease.

At LH_2_ temperature, the LVDG sensor has characteristics of the ideal L-V sensor discussed above. The small size and large d*R*/d*T* of LVDG, Fig. 6, give it both a fast response and a large signal change when it is powered by a constant current. At 9 mA, the voltage signal ratio *V*_l_/*V*_g_=7. Thus the power dissipated in the gas 1/7 is that dissipated in the liquid. This inherent decrease in power reduces *T*_g_ − *T*_l_ which reduces the response time. Even at 20 mA where the sensor is insulated by the formation of a vapor layer, the signal ratio is still 2.7 and the time constants are nearly the same as at 9 mA. No advantage is achieved at the higher current and the disadvantages of a noisy signal and higher power into the LH_2_ make higher currents unattractive.

Most of the other sensors tested did not have large values of d*R*/d*T* at LH_2_ temperatures, hence their signal ratios were modest compared to LVDG. Many did respond rapidly when entering the liquid. Often this was true because the power to the sensor was too low which caused a slow response upon withdrawal to the gas. To speed their response leaving the liquid, a higher sensor power was needed. Since a higher sensor power raises the liquid entry response time especially when d*R*/d*T* is small, smaller size is the only way to improve response time.

The active portion of many of these sensors was small with respect to their overall size. Applying sufficient power to rapidly remove the liquid film risks destroying the sensor. The long equilibrium time on the liquid to gas transition of some of the sensors probably came from the large heat capacity of the sensor. A sensor that can withstand a higher power could be driven by a logic controlled power supply that decreases sensor power as soon as the L-V sensor begins responding to gas. This would reduce heating of the sensor in the gas, which shortens the response time entering the liquid. The magnitude of *S*_l_ − *S*_g_ is decreased by this approach, however.

At LN_2_ temperature, LVDG still showed a relatively square signal pulse for a transition from liquid to gas to liquid, but the smaller d*R*/d*T* resulted in a greatly reduced ratio of *V*_l_/*V*_g_. The sensor power in liquid was about the same as that for the LH_2_ tests. The response times are about 0.1 for both, 50 times longer than found for LH_2_. The long response time entering the liquid is caused by a large total sensible heat content of the sensor between *T*_l_ and *T*_g_.

The large change in sensor resistance and the fast response time make the LVDG sensor the best of those tested for use in LH_2_. Of the sensors tested, LVDG had the fastest response in LN_2_ for withdrawal from the liquid. The response was slower and the signal level change reduced from that in LH_2_. Possibly a stronger signal could be obtained for LVDG in LN_2_ if the doping level of the silicon sensor was increased. The sensor has the disadvantage in that it is not commercially available at present.

These tests have shown that sensor lead orientation relative to the liquid surface can be important to the response time of the liquid to gas transition. The different behavior for the two lead geometries of LVDG in LH_2_ and PT1 in LN_2_ illustrates this. Liquid draining down the leads from crevices in the insulation and lead anchoring hardware probably caused delays in the sensor response. Bending the lead wires into a *J* shape, with the sensor on the short leg, might eliminate any flow down the leads to the sensor. The orientation effect observed in this work was caused by gravity driven flows. In zero gravity, the lead orientation with respect to the direction of motion of the liquid surface could still affect the response time. Changing the direction of the leads relative to the surface could cause a variation in the amount of liquid remaining in the sensor.

Since the sensors and their supports will always have a finite size, splashing will occur when the liquid surface moves by. Smaller sensors should generate less surface disturbance. The support structure for the sensors may be a much stronger source of disturbance. A large amount of splashing and bubble introduction occurred during these tests, but the switching of the sensors between states tended to be fairly independent of other disturbances.

Table 3 shows the best operating power found during these tests for each of the sensors and the resulting signal ratio and time constants. The optimum performance of a sensor might be expected to occur at the power level that gave equal time delays for entering and leaving the liquid. This was the case for sensors tested at high power. For some sensors, in particular the diode sensors, this power was not reached.

It should be emphasized that the commercially available sensors tested here are meant to be thermometers and not liquid-vapor sensors. These sensors were not designed to be used as they were used in these tests. The results given here do not reflect in any way upon the ability of these sensors to measure temperature. Only LVDG was specifically designed to be a liquid-vapor detector.

## Figures and Tables

**Fig. 3a:**
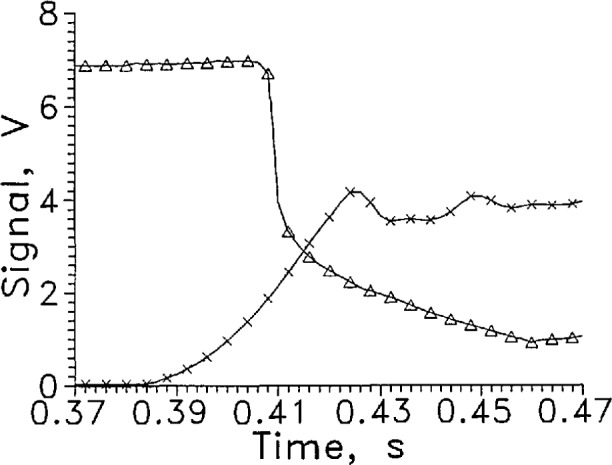
Output signal from LVDG at a transition from LH_2_ to GH_2_, Δ. Data of Fig. 2 on an expanded time scale. Position sensor voltage, ×. Leads horizontal, *I* = 9 mA, alternate data points shown.

**Fig. 3b:**
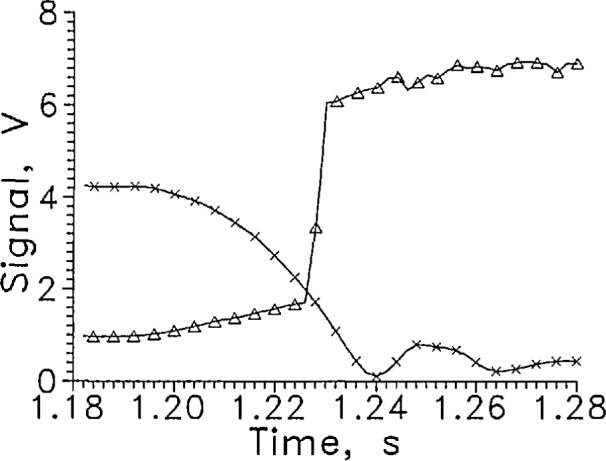
Output signal from LVDG at a transition from GH_2_ to LH_2_, Δ. Data of Fig. 2 on an expanded time scale. Position sensor voltage, ×. Leads horizontal, *I* = 9 mA, alternate data points shown.

**Fig. 8a:**
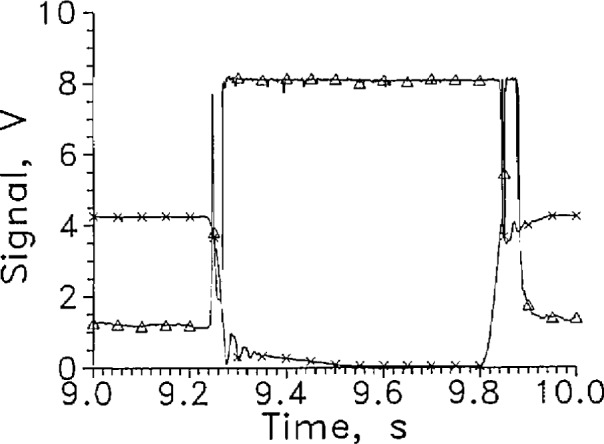
Output signal from LVDG for one cycle between LH_2_ and GH_2_, Δ. Position sensor voltage, ×. Leads vertical, *I=9* mA, every 50th data point shown.

**Fig. 8b:**
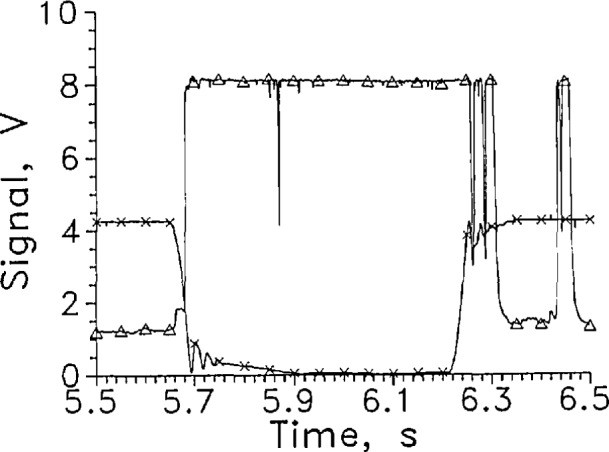
Output signal from LVDG for one cycle between LH_2_ and GH_2_, Δ. Position sensor voltage, ×. Leads vertical, *I* = 9 mA, every 50th data point shown.

**Table 1:** Sensors tested as liquid-vapor surface sensors

Sensor designation	Description	Dimensions	Leads	Support
LVDG	Bulk doped silicon resistor	Cube 1/4 mm	0.05 ram SS	Leads
SOS	Silicon resistor on sapphire	Rectangular 2.8 × 1.9 × 0.25 mm	0.825 mm	SS Wire
K	Silicon resistor on sapphire	Rectangular 1.5×0.5×0.013 mm	Unknown	0.05 mm SS
PT1	Platinum on ceramic	Rectangular 1×4×5 mm	AuPd alloy	Leads
PT2	Platinum on ceramic	Rectangular 1×2×2.3 mm	AuPd alloy	Leads
DTO	Encapsulated Si diode	Cylinder 1.45 mm dia. 3.2 ram long	Unknown	Leads
DT1	Encapsulated Si diode	Rectangular 3.2×1.9×1 mm	Au plated Kovar	Leads
S	Si diode on sapphire	Rectangular 2×2×0.15 mm	Phosphor bronze	Leads
Mdac	10 kΩ carbon resistor +100 Ω nominal thermistor	Irregular 15×33×5 mm	Brass heat sink	Heat sinks
Carbonl	920 Ω, 1/8 W resistor		Copper	Leads

**Table 2:** LVD Goddard response time in LN_2_

Current (mA)	Power in liquid (mW)	Avg. time constant for liquid entiy (ms)	Avg. time constant for liquid exit (ms)	Sum (ms)	Relative holder position
40	64	57	154	211	High
40	64	48	308	356	Low
50	100	118	101	219	High
50	100	106	107	213	Low

**Table 3:** Operating parameters for best performance

Sensor series *R*	*V* or *I*	Power liquid	Power, vapor	Max *S*_l_/*S*_g_	Avg. response to liquid	Avg. response to gas	Comments
Hydrogen							

LVDG	9 mA	63 mW	9mW	7	1.5 ms	3.5 ms	1
PT1 46 Ω	20 V	2W	0.8 W	19	0.3 s	0.4 s	2, 3
PT2 29 Ω	12 V	1W	1.3 W	50?	0.25 s	0.6 s	2,3,4
DT1	40 mA	120 mW	110 mW	1.1	<2ms	0.9 s	4
S	40 mA	120 mW	100 mW	1.2	<2ms	0.4 s	
Carbonl 200 Ω	20 V	0.3 W	0.4 W	1.25	60 ms	0.5 s	
Mdac 1000 Ω	100 V	0.36 W	0.5 W	1.4	<1s	2s	

Nitrogen							

LVDG	50raA	175 mW	125 mW	2	0.11s	0.11s	2
PT1 100 Ω	20 V	0.84 W	0.5 W	>2	1.4 s	0.6 s	1
PT2 29 Ω	10.5 V	0.8 W	0.5 W	>4	1 s	2.1s	3
DT1	40 mA	0.1 W	0.1 W	~1.05	~10 ms	~10 s	
S	40 mA	0.1 W	0.1 W	~1.05	~10 ms	~2 s	
Carbon1 200 Ω	30 V	1.3 W	1.3 W	1.11	60 ms	0.5 s	
Mdac 1000 Ω	100 V	0.5 W	1.3 W	1.25	~0.5 s	~2 s	
K	5 mA	33 mW	29 mW	1.16	~1 s	~1 s	

1 Liquid to gas response time increases for vertical leads.

2 Maximum *S*_l_/*S_g_* is estimate for a four lead measurement.

3 Tested only with leads vertical.

4 Did not always make transition to gas reading on rapid cycling.

**Table 4:** PT1 test results in LN_2_

Applied volts	Delay in liq. (s)	Range (s)	Delay out liq. (s)	Range (s)	*S*_l_/*S*_g_ power	Max (W)
12	0.05	0.01 to 0.17	3.9	1.9 to 6.6	>1.7	0.3
15	0.17	0.07 to 0.29	2.5	1.7 to 3.3	>1.7	0.5
20	0.6	0.47 ta 0,63	1.4	1 to 1.8	>2	0.8

## References

[1] Burgeson D, Richards R (1966). Test results of liquid level point sensor operation in liquid hydrogen.

[2] Burgeson D, Richards R (1965). Selecting liquid level transducers for cryogenic service.

[3] Burgeson D, Pestalozzi W, Richards R (1964). The performance of point level sensors in liquid hydrogen, Advances. Cryogenic Engineering.

[4] Siegwarth J, Voth R, Snyder S (1992). Liquid-vapor surface sensors for liquid nitrogen and hydrogen. Cryogenics.

[5] DiPino M, Serlemitsos S (1990). Discrete liquidAapor detectors for use in liquid helium, Advances. Cryogenic Engineering.

